# Effect of Aronia Extract on Collagen Synthesis in Human Skin Cell and Dermal Equivalent

**DOI:** 10.1155/2022/4392256

**Published:** 2022-08-08

**Authors:** Hwa-Rim Lee, Hye Guk Ryu, Yunji Lee, Ju An Park, Seongju Kim, Chang Eon Lee, Sungjune Jung, Kyung-Ha Lee

**Affiliations:** ^1^Department of Materials Science & Engineering, Pohang University of Science and Technology (POSTECH), 77 Cheongam-Ro, Nam-Gu, Pohang 37673, Republic of Korea; ^2^Division of Cosmetic Science and Technology, Daegu Haany University, Hanuidae-ro 1, Gyeongsan, Gyeongbuk 38610, Republic of Korea; ^3^Department of Mechanical Engineering, Pohang University of Science and Technology (POSTECH), 77 Cheongam-Ro, Nam-Gu, Pohang 37673, Republic of Korea; ^4^Department of Molecular Biology, Pusan National University, 2, Busandaehak-ro 63beon-gil, Geumjeong-gu, Busan 46241, Republic of Korea

## Abstract

The regulation of collagen synthesis, which occurs in fibroblasts in the dermal layer, is a key process in dermis regeneration and skin reconstruction. Herein, we investigated whether *Aronia melanocarpa* extract affects the human skin condition. We focused on type I collagen synthesis using two different types of model systems: a monolayer of cells and a bioprinted 3D dermal equivalent. The Aronia extract showed no cytotoxicity and increased cell proliferation in neonatal human dermal fibroblasts. Treatment with Aronia extract increased the transcription of *COL1A1* mRNA in direct proportion to the extract concentration without causing a decrease in *COL1A1* mRNA degradation. Additionally, the Aronia extract inhibited the expression of MMP1 and MMP3, and an increase in type I collagen was observed along with a decrease in MMP1 protein. We also fabricated dermal equivalents from type I collagen (the major component of the dermis) and dermal fibroblasts by bioprinting. In the 3D dermis model, the compressive modulus directly affected by collagen synthesis increased in direct proportion to the Aronia extract concentration, and expression levels of *MMP1* and *MMP3* decreased in exactly inverse proportion to its concentration. The findings that the Aronia extract increases synthesis of type I collagen and decreases *MMP1* and *MMP3* expression suggest that this extract may be useful for the treatment of damaged or aged skin.

## 1. Introduction

The natural extract contains substances with various biological activities and has been used in many fields such as medicine [1], agriculture [2], and food science [3]. There have been steady attempts to obtain extracts in their natural state and utilize them in various fields such as disease treatment and symptom improvement [4, 5]. A special focus has been on berry species because berry crops are well-recognized as a valuable source of bioactive chemicals that can be employed for health-promoting activities [6]. Moreover, berry species are already widely consumed as food and can be easily used for the treatment of diseases with simple processing due to concerns about toxicity [7–9].

*Aronia melanocarpa*, also known as black chokeberry, is widely consumed as a food and also used for various purposes such as natural food colorants and herbal medicine [10, 11]. It is valued for its potential antiaging effects because it contains many phenolic compounds [12, 13]. Several studies showed that Aronia exhibits diverse biological properties, including antioxidant [14, 15], anti-inflammatory [16], and anticancer [17] effects in mammalian cells. The role of Aronia extract in promoting the health of arteries [18], liver [15, 19], intestine [20], and brain [21] has been reported continuously.

Nevertheless, only a few effects of the Aronia extract have been reported on skin: enhancement of proinflammatory response in HaCaT cells [22] and recovery of UV damage in mouse skin [23]. Thus, further research is needed to determine the antiaging effect of Aronia extract on the skin. Aging is associated with changes in collagen fiber content in the skin, primarily reduced type I collagen synthesis, which contributes to deep wrinkles and the sagging of skin [24, 25]. These processes are prominent in the fibroblast and dermal layers of the skin [26, 27]. Skin aging is also associated with increased expression of degradative enzymes such as matrix metalloproteinase (MMP). It can break down collagen and elastin fibers, resulting in reduced collagen deposition [28].

Herein, we investigated the MMP-mediated changes in dermal equivalent after treatment with Aronia extract. To experimentally verify our results in human tissue models, we analyzed cell proliferation, gene expression, and promoter assay by Aronia extract in primary dermal fibroblasts and monitored how MMP-1 and MMP-3, which are directly involved in collagen degradation, change according to the treatment of Aronia extract. In addition to the monolayer of cells, we used 3D-printed dermal equivalents, and its results were interpreted by linking molecular analysis with mechanical properties that represent physical changes in human skin.

## 2. Methods

### 2.1. Preparation of Aronia Extract

Aronia extract (Danjoungbio, Gangwon-do, Korea) was purchased and used at the indicated concentrations. According to the manufacturer's instructions, Aronia extract was prepared from fruit of *Aronia melanocarpa* berries using 70% ethanol.

### 2.2. Cell Culture

Human epidermal keratinocytes (HaCaT cells) were grown in Dulbecco's modified Eagle's medium (DMEM, HyClone, UT, USA) supplemented with 10% heat-inactivated fetal bovine serum (HyClone), 100 units/ml penicillin, and 100 *μ*g of streptomycin (HyClone). HaCaT cell was subcultured to reach approximately 80%–90% confluence. Neonatal human dermal fibroblasts (HDFn cells; Gibco, NY, USA) were grown in fibroblast expansion basal medium (Gibco) with a low serum growth supplement (LSGS) kit (Gibco) consisting of 2% fetal bovine serum, 1 *μ*g/ml hydrocortisone, 10 ng/ml human epidermal growth factor (EGF), 3 ng/ml basic fibroblast growth factor (FGF), 10 *μ*g/ml heparin, and gentamicin/amphotericin B solution (10 *μ*g/ml and 0.25 *μ*g/ml, respectively). HDFn cell was subcultured to reach approximately 90% confluency at passage 5 or lower.

### 2.3. Plasmid Construct and Drug Treatment

A promoter-reporter plasmid for human *COL1A1* (GeneCopoeia, MD, USA) containing the human *COL1A1* promoter and *Gaussia* luciferase was used in this study. In the *COL1A1*3′UTR-expressing plasmid, *Renilla* luciferase was fused with full-length human 3′UTR of *COL1A1*, and firefly luciferase was used as an internal control [29]. psiCHECK2-*COL1A1* full-length 3′UTR was a gift from Joan Massague (Addgene plasmid # 26993; http://n2t.net/addgene:26993; RRID: Addgene_26993). Reporter plasmids were transfected into HDFn cells using the Neon® transfection system (Thermo Fisher Scientific). To block gene transcription, 24 h incubated transfected cells were treated with 5 *μ*g/ml actinomycin D (Sigma; Cat.# A9415) and then harvested at the indicated time points.

### 2.4. Cell Viability Assessment

Cells were seeded into 96-well plates at a density of 5 × 10^4^ cells per well and incubated overnight. Cell viability was determined after 12–72 h in the presence or absence of Aronia extract using an MTT assay or a Cell Counting Kit-8 (Dojindo, Kumamoto, Japan). Plates were read using an Infinite M Nano microplate reader (Tecan, Zurich, Switzerland), according to the manufacturer's instructions.

### 2.5. RNA Isolation and cDNA Synthesis

RNA isolation from skin cells, followed by cDNA synthesis, was performed as described previously [30, 31]. Briefly, cells were lysed with TRIzol reagent (Thermo Fisher Scientific, Waltham, MA, USA), according to the manufacturer's instructions, and total RNA was extracted. The yield and purity of RNA were determined using the Infinite M Nano microplate reader (Tecan). Then, 1 *μ*g of total RNA from each sample was reversely transcribed using GoScript™ Reverse Transcription Mix, Oligo(dT) (Promega, WI, USA), according to the manufacturer's instructions.

RNA isolation from dermal equivalents, followed by cDNA synthesis, was carried out as described previously [32]. In brief, total RNA was extracted from Aronia extract-treated tissues using RNeasy Mini Kit (Qiagen, Germany). The yield and purity of the isolated total RNA were measured using the NanoDrop® spectrophotometer (Thermo Fisher Scientific). Then, 1 *μ*g of each total RNA sample was reversely transcribed using a High Capacity cDNA Reverse Transcription Kit (Applied Biosystems, CA, USA), according to the manufacturer's instructions.

### 2.6. Quantitative Real-Time PCR (qRT-PCR)

The cDNA levels of endogenous genes in skin cells were determined by performing qRT-PCR using a QuantStudio 3 Real-Time PCR System (Thermo Fisher Scientific) with the TaqMan Universal PCR Master Mix (Thermo Fisher Scientific), according to the manufacturer's instructions. The following amplification program was used: polymerase activation at 95°C for 10 min, 40 repeated cycles of 95°C for 15 s, and 60°C for 1 min. The cDNA levels of endogenous genes in dermal equivalents were detected by using a StepOne Plus Real-Time PCR System (Applied Biosystems) with SYBR Green master mix (Applied Biosystems). Melting curves were generated to validate the PCR process after each amplification. Primers used for qRT-PCR are as follows: *COL1A1*, 5′-ATGTGCCACTCTGACTGGAA-3′ and 5′-CTTGTCCTTGGGGTTCTTGC-3′; *MMP1*, 5′-GCATATCGATGCTGCTCTTTC-3′ and 5′-GATAACCTGGATCCATAGATCGTT-3′; *MMP3*, 5′-CTGGAGATTTGATGAGAAGA-3′and 5′-CCAACTGTGAAGATCCAGTA-3′; *RPL32*, 5′-TATTGGCAACGAGCGG-3′ and 5′-CGGATGTCAACGTCAC-3′; and *GAPDH*, 5′-CGTAGCTCAGGCCTCAAGAC-3′ and 5′-GCTGCGGGCTCAATTTATAG-3′.

### 2.7. Immunoblot Analysis

Immunoblot analyses were performed using monoclonal anti-*β*-actin (sc-47778, Santa Cruz Biotechnology), polyclonal anti-collagen I (ab34170, Sigma-Aldrich), and monoclonal anti-MMP-1 (ab134184) as primary antibodies. Horseradish peroxidase-conjugated species-specific secondary antibodies (Thermo Fisher Scientific) were visualized using Pierce™ ECL Western Blotting substrate (Thermo Fisher Scientific) under FUSION Solo S (Vilber, Lourmat, France). The acquired images were analyzed according to the manufacturer's instructions.

### 2.8. Preparation of Bioink for the Dermis Model

The dermal layer matrix was prepared by applying the microextrusion-based method, with slight modifications, to generate dermal equivalent, as described previously [33]. In brief, type I collagen (Dalim Tissen, Republic of Korea) was dissolved in 0.1% acetic acid to obtain a 0.75% (*w*/*v*) acidic aqueous solution. Then, 10X DMEM/F-12 mixture (Gibco), 100 units/ml penicillin, and 100 *μ*g of streptomycin (HyClone) were added to the 0.75% (*w*/*v*) acidic aqueous solution to support cell proliferation in the dermal layer matrix. The acidic collagen solution was neutralized by adding a 10X reconstitution buffer consisting of NaOH, NaHCO_3_, and HEPES, and the final collagen concentration was adjusted to 0.6% (*w*/*v*). HDFn cells were harvested using trypsin/EDTA (Gibco) and trypsin neutralizer (Gibco) solution to construct the dermis model. Then, bioink (2.5 × 10^5^ viable cells/ml) was subjected to bioprinting (described below).

### 2.9. Bioprinting, Tissue Culture, and Aronia Extract Treatment

First, 800 *μ*l of 0.6% collagen with 2.0 × 10^5^ HDFn cells was directly dispensed into a 12 mm Transwell® with a 3.0 *μ*m pore polycarbonate membrane insert (Corning, NY, USA) using a pneumatic extrusion printer (350 PC, Musashi Engineering, Inc., Japan), equipped with a 24-G needle (Musashi Engineering, Inc.), at a pressure of 10 kPa. The printing product was stored at 4°C before dispensing. Microextrusion was carried out first at room temperature and then at 37°C in 5% CO_2_/95% air incubator until crosslinking. Then, dermal culture medium (fibroblast expansion basal medium with LSGS) was added to the crosslinked printed product to begin submerged culture. Half of the medium was changed every 2 or 3 days. After 7 days of dermal layer culture, the 3D dermis model was treated at the indicated concentrations of Aronia extract for 72 h.

### 2.10. Compressive Modulus of Aronia Extract-Treated Dermal Layer Matrix

The test was conducted to determine the compressive modulus of Aronia extract-treated 3D dermis model (*n* = 3) of approximately the same diameter (12 mm). In axial compression test mode, the compressive modulus of dermal layer matrices was analyzed on a rheometer (Discovery HR 20, TA Instruments, DE, USA). The disc-shaped dermal layer matrices were positioned between the bottom plates (25°C) and parallel spindle (20 mm diameter) of the rheometer. Compressions were applied at a constant rate of 20.0 *μ*s/s for 500 s. The compressive modulus of each sample was calculated from the linear region of the slope on the strain-stress curve.

### 2.11. Immunohistochemical Analysis

Immunohistochemistry was carried out using cross-sectioned tissues as previously described [33]. Briefly, Aronia extract-treated 3D dermis models were dehydrated 5 min and then fixed in 4% paraformaldehyde solution (Biosesang, Republic of Korea). Fixed tissues were transferred to frozen section compound (Leica Biosystems, Deutschland) to generate cryomolds. A 20 *μ*m thick sectioned tissue was obtained using a cryotome (CM1860, Leica Biosystems). The cross-sectioned tissues were stained with anti-type I collagen (Abcam, UK) with Alexa® 488-conjugated goat anti-rabbit IgG (Invitrogen, CA, USA). To visualize cell nuclei in tissues, Hoechst 33342 (Invitrogen) staining was applied.

### 2.12. Statistical Analysis

All comparisons between groups were performed by one-way analysis of variance (ANOVA): ∗, *p* < 0.05; ∗∗, *p* < 0.01, ∗∗∗, *p* < 0.005, ∗∗∗∗, *p* < 0.0001; and n.s., not statistically significant. All assays were representative of at least three separately repeated experiments.

## 3. Results

### 3.1. Aronia Extract Moderately Increases the Growth of Human Skin Cells

In order to determine whether the Aronia extract exerts cytotoxic effects on skin cells, the normal human keratinocytes (HaCaT cells) and the neonatal human dermal fibroblasts (HDFn cells) were exposed to various concentrations of the Aronia extract, and cell viability was assessed.

**Figure 1:**
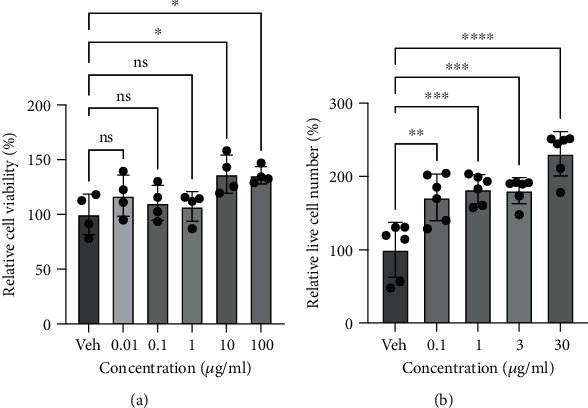
Effect of Aronia extract on cell viability on human epidermal keratinocytes (HaCaT cells) and dermal fibroblasts (HDFn cells). (a) Cytotoxic effects on HaCaT cells. The HaCaT cells were treated with the indicated concentrations (Conc) of the Aronia extract for 24 h and then subjected to the MTT assay. The viability of the vehicle was 100 (*n* = 4). (b) Cytotoxic effects on HDFn cells. The HDFn cells were treated with the indicated concentrations of the Aronia extract for 72 h and then subjected to the CCK-8 assay. The value of the vehicle (Veh) was 100 (*n* = 6).

Treatment with 0.01–100 *μ*g/ml Aronia extract for 24 h resulted in cytotoxicity to HaCaT cells was not observed, and an increase in cell proliferation was observed in the presence of 10 or 100 *μ*g/ml Aronia extract (Figure 1(a)). In order to verify the effect of Aronia extract on cytotoxicity in HDFn cells, it was exposed to Aronia extract for 72 h. Aronia extract induced the proliferation of HDFn cells in a dose-dependent manner (Figure 1(b)). Thus, these results suggest that Aronia extract does not exhibit cytotoxicity; rather, it promotes the growth of human skin cells.

### 3.2. Aronia Extract Increases *COL1A1* mRNA Expression in Human Dermal Fibroblasts

**Figure 2:**
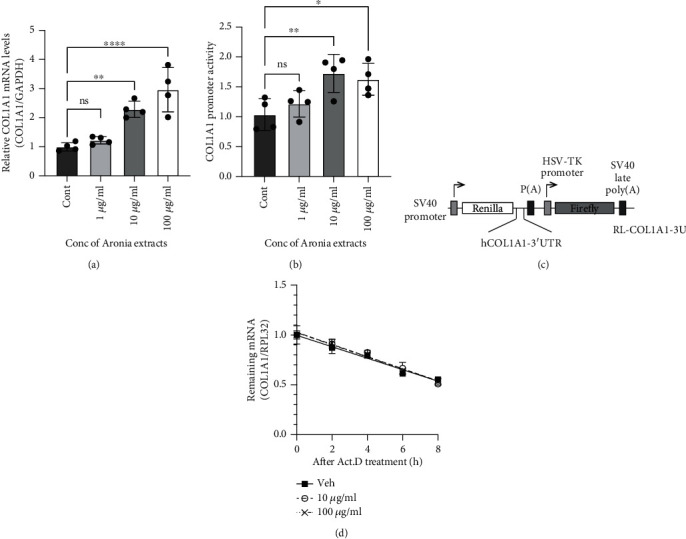
Aronia extracts promote collagen synthesis in human dermal fibroblasts. (a) Expression analysis of *COL1A1* in HDFn cells. The HDFn cells were treated with the indicated concentration of Aronia extract for 48 h. Then, relative *COL1A1* mRNA levels were measured by qRT-PCR using the indicated gene-specific primers. Bars represent the mean ± standard error of the mean (*n* = 6). Control (Cont). (b) Aronia extract elevated *COL1A1* promoter activity. Luciferase reporter assays were performed in HDFn cells (*n* = 6). (c) Schematic representation of reporter gene constructs. Reporter construct composed of the psiCHECK2-backbone plasmid containing the *Renilla* luciferase gene and human *COL1A1* 3′UTR (RL-COL1A1-3U). (d) *COL1A1* mRNA levels in HDFn cells treated with actinomycin D (Act.D) for 2, 4, 6, and 8 h. *Y*-axis shows the relative levels of *COL1A1* mRNA (*n* = 3).

First, we investigated the effect of Aronia extract on collagen synthesis. Cells were treated with the indicated concentrations of Aronia extract (0, 1, 10, or 100 *μ*g/ml) for 48 h, and the level of *COL1A1* mRNA was determined by qRT-PCR. Treatment with 10 or 100 *μ*g/ml Aronia extracts increased *COL1A1* expression in HDFn cells (Figure 2(a)), demonstrating that Aronia extract regulates *COL1A1* in HDFn cells. To further investigate the effect of Aronia extract on the transcriptional activation of *COL1A1*, we transfected HDFn cells with a *COL1A1* promoter-reporter vector. We confirmed that Aronia extract treatment upregulated the level of *COL1A1* promoter activity (Figure 2(b)). To examine the further role of Aronia extract in *COL1A1* mRNA expression regulation, the stability of *COL1A1*3′UTR (reporter) was measured upon treatment with actinomycin D, which inhibits transcription. The results showed that the half-life of *COL1A1*3′UTR was not affected by Aronia extract (Figure 2(d)), indicating that Aronia extract regulates *COL1A1* mRNA level not by mRNA degradation but by transcriptional activation.

### 3.3. Effect of Aronia Extract on *MMP1*, *MMP3*, and Type I Collagen Expressions in Human Dermal Fibroblasts

**Figure 3:**
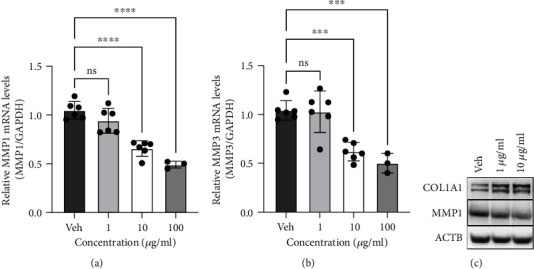
Aronia extracts downregulate MMPs. (a and b) *MMP1*(a) and *MMP3*(b) expression levels in HDFn cells. The HDFn cells were treated with the indicated concentration of Aronia extract for 24 h, and *MMP1* and *MMP3* mRNA levels were measured by qRT-PCR using the indicated gene-specific primers. The value of the vehicle was 1 (*n* = 6). (c) Immunoblots for COL1A1 and MMP-1 in HDFn cells treated with Aronia extracts. *β*-Actin (ACTB) was a loading control. Aronia extracts increased COL1A1 protein levels and decreased MMP-1 protein levels in HDFn cells.

To clarify whether Aronia extract regulates the expression of MMPs, we measured the mRNA levels of *MMP1* and *MMP3*. The results showed that the expression of *MMP1* and *MMP3* decreased following treatment with Aronia extract (Figures 3(a) and 3(b)). Furthermore, type I collagen and MMP-1 protein levels were changed controversially with the concentration of Aronia extract treated (Figure 3(c)). These results suggest that Aronia extract plays a role at the same time for type I collagen and MMP-1, which is known to degrade it.

### 3.4. Effect of Aronia Extract on Material Properties of the 3D Dermis Model

**Figure 4:**
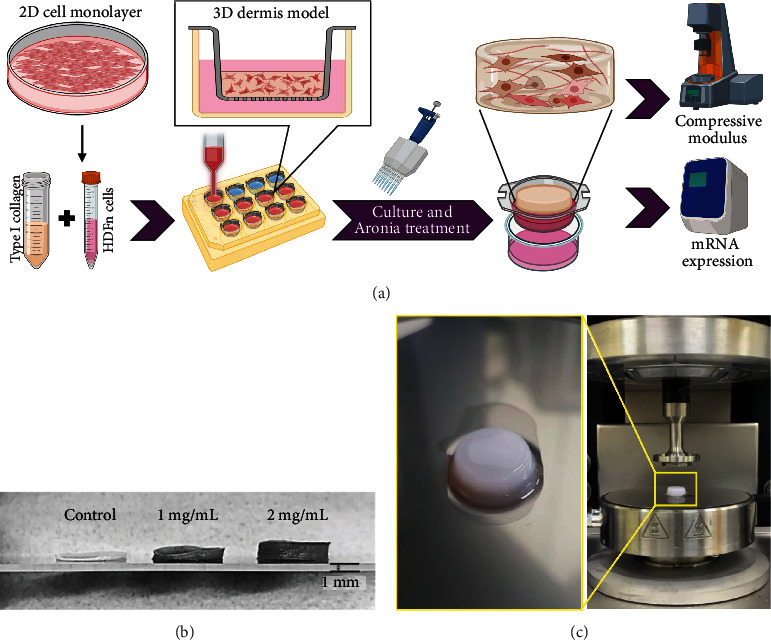
Scheme of experimental procedure and photo image of 3D model. (a) 2D cell monolayer culture and 3D dermis model fabrication process using microextrusion printing (created with http://biorender.com/). (b) Volume change after treatment with excess Aronia extract. (c) Photo image of 3D model (left) and experimental photo image during compression test measurements using rheometer (right).

To investigate whether the Aronia extract affects collagen synthesis not only in the 2D cell culture condition which is a monolayer of cells but also in our body, we introduced the bioprinting method to create a 3D dermis model of collagen-containing dermal fibroblasts (Figure 4(b)). To observe the direct effect of the Aronia extract on the 3D dermis model, we measured the change in tissue volume upon treatment with different concentrations of the Aronia extract. In the situation where a slight excess of Aronia extract was administered (0, 1, or 2 mg/ml) to see a clearer correlation, the 3D dermis model showed a dramatic volume change with higher concentrations of Aronia extract, showing a positive correlation (Figure 4(b)).

**Figure 5:**
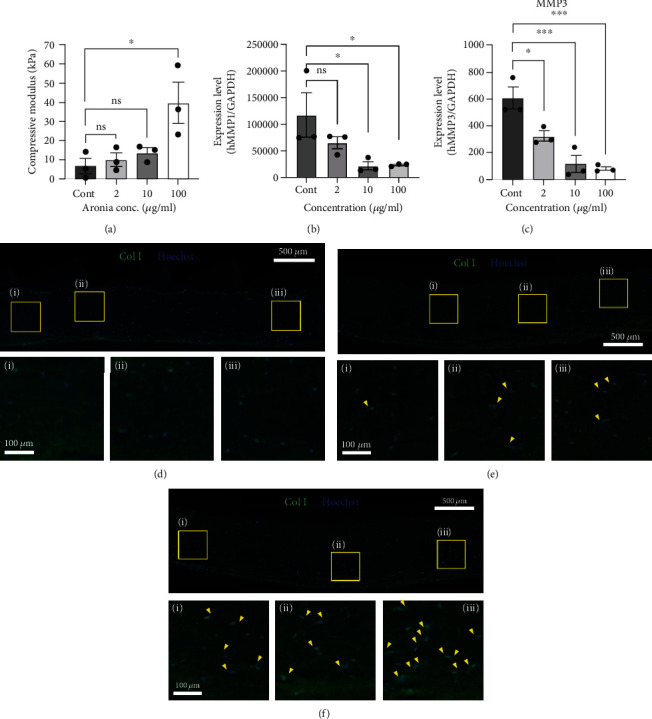
Aronia extract regulates the material property and MMP expressions in the 3D dermis model. (a) Compressive modulus of the dermal layer matrix following treatment with Aronia extract at different concentrations (*n* = 3). (b and c) Transcript levels of *MMP1* (b) and *MMP3* (c) in dermal layer matrices treated with the indicated concentration of Aronia extract for 72 h. mRNA levels were measured by qRT-PCR using the indicated gene-specific primers (*n* = 3). (d–f) Immunohistochemical staining using type I collagen antibody (anti-Col1) in dermal equivalent after Aronia extract treatment in the concentration of 0 *μ*g/ml (d), 10 *μ*g/ml (e), and 100 *μ*g/ml (f). Arrowheads indicate the distribution of type I collagen generated around the dermal fibroblasts.

Additionally, to identify the effect of Aronia extract on the mechanical properties of the tissue, we measured the compressive modulus of the dermal model treated with various concentrations (0, 2, 10, or 100 *μ*g/ml) of Aronia extract (Figure 4(c)). The compressive modulus of the dermal model increased with the increase in Aronia extract concentration, with values of 6.72 ± 3.98, 9.96 ± 3.43, 13.61 ± 2.40, and 39.63 ± 10.54 kPa at 0, 2, 10, or 100 *μ*g/ml Aronia extract, respectively (Figure 5(a)).

### 3.5. Effect of Aronia Extract on MMP Expressions and Type I Collagen Synthesis in the 3D Dermis Model

To confirm that the volume increase and mechanical property change observed in the 3D dermal model treated with Aronia extract was caused by the inhibition of collagen degradation at the molecular level, we measured the expression of *MMP1* and *MMP3* in the dermis model (Figures 5(b) and 5(c)). Similar to the results obtained in the monolayer of cells, the expression of *MMP1* and *MMP3* also reduced in the 3D model after Aronia extract treatment. This reduction in expression was in inverse proportion to the concentration of the Aronia extract. These results suggest that Aronia extract is involved in collagen synthesis through the MMP-mediated collagen inhibition processes.

Next, to confirm whether this effect led to actual collagen synthesis, the expression of type I collagen was validated in the tissue (Figures 5(d)–5(f)). In the 3D dermis model treated with Aronia extract at concentrations of 0, 10, and 100 *μ*g/ml, we did not observe a distinct difference in the expression of type I collagen throughout the tissues. However, we confirmed that as the concentration of the treated Aronia extract increased, the amount of type I collagen newly generated around dermal fibroblasts also increased (Figures 5(e) and 5(f)).

## 4. Discussion

Identification of the interaction between collagen and MMPs at the molecular level is important for understanding the process of responding to external stimuli, especially in the skin [34–36]. Therefore, to find new antiaging agents that affect the regeneration of the dermal layer, many studies have focused on collagen–MMP interactions in fibroblasts [27, 28]. However, interactions revealed using nonhuman mammalian models such as mice and rats are limited, as they do not apply to real human tissues. On the other hand, data obtained from human tissue biopsy makes it difficult to exclude the influence of other MMP-responsive cells and the crosstalk among various MMP-regulated ECM components such as type II, IV, and IX collagen [37], fibronectin [38], and laminin [35]. The method of separating and culturing only the target cell allows the analysis of the interaction between a specific MMP and its target collagen; however, this method also has limitations in terms of other 2D environments and the lack of an extracellular environment.

The data presented here demonstrate what happens in primary human cells at the molecular level and selectively represent only the interaction of fibroblasts with type I collagen, which is the major component of the human dermis. Considering that molecular interactions related to MMPs, which have been mainly revealed in 2D cell culture conditions from previous studies, act complexly in a 3D environment and make it difficult to predict actual physiological changes, we highlight the advantages of the method used in this study. In particular, we showed that Aronia extract is independent of COL1A1 mRNA degradation (Figure 2); therefore, the elimination of the regulation that arises at the posttranscriptional level underscores the role of the Aronia extract. These results indicate that Aronia extract is potentially associated with changes in age-related gene expression [39] and suggest that this extract can be an important component of antiaging skin products.

From an experimental point of view, we treated the 3D model with a slight excess of the Aronia extract compared with the commonly processed extract. However, in the field of pharmacology, considering that the drug efficacy in an actual 3D tissue model is slightly lower than that in the existing 2D cell culture environment, this could be an acceptable result. Rather, we could observe that the Aronia extract was involved in MMP-1- and MMP-3-mediated collagen regulation (Figures 3 and 5) while maintaining high cell viability even at high concentrations (100 *μ*g/ml) (Figure 1). This implies that the Aronia extract can be applied at a rather high concentration with low toxicity, unlike many new candidates that show cytotoxic effects even at low concentrations and cause a sharp decrease in cell viability at concentrations above a certain threshold.

This study also suggests the significance of the candidate test procedure supported in 3D microenvironments and provides new information. We introduced bioprinting to produce reliable 3D tissue models, which reduced sample variation commonly found when building collagen-based dermal equivalent structures [40, 41]. In the fabrication of 3D skin models, it is widely used the tissue culture insert to support an air-liquid interface to the tissue [42, 43]. When skin cells are embedded within collagen matrix and released by manual methods such as pipetting into culture inserts, it is difficult to control the volume released due to the viscosity of collagen and handling error. In addition, the tip (or nozzle) does not have vertical access to the culture insert in the manual process, resulting in an asymmetric meniscus during the gelation. This makes the large variation between samples and seriously causes experimental errors in the measurement process of mechanical properties in this study. On the other hand, bioprinting technique inherently guarantees reproducibility and precise control, which can contribute to the problems described, especially to the reduction of sample-to-sample variation and experimental errors. Consequently, it allowed us to observe the expression of MMP, which reversely responded to Aronia extract treatment in a dose-dependent manner even in the 3D tissue sample, containing only a relatively small number of cells, compared with the monolayer of cells (Figures 5(b) and 5(c)). Together with this, we were able to observe type I collagen production near the cells responded to Aronia extract treatment in a dose-dependent manner (Figures 5(e) and 5(f)). These results are consistent with the results of ECM protein analysis in 3D mass [44].

Moreover, the treatment of 3D tissue with Aronia extract increased the elasticity of whole tissues directly related to skin regeneration in a dose-dependent manner. This is consistent with the results of multiple studies focused on the mechanical properties of human tissues [26, 45]. In the current study, the MMP-1- and MMP-3-mediated effects of Aronia extract showed a similar pattern on gene expression and the compressive modulus of the whole tissue, suggesting that the intracellular molecular response also affects macrostructure properties through cell–ECM interaction. Thus, we could present a methodology that leads to molecular and cellular analysis, human tissue model fabrication and culture, and response in tissue property for test methods that utilize the natural extract as a novel candidate.

Based on this approach, we expect that additional studies will be performed to examine (1) a full-layered structure containing epidermal layers, (2) Aronia extract-induced physical changes in skin containing microstructures such as papilla, (3) the response to external stimuli and recovery by Aronia extract, and (4) the effect of a single compound isolated from the Aronia extract on collagen synthesis. Given the well-established screening methodologies and the broad applicability of bioprinting techniques, these different approaches deserve further investigation.

## 5. Conclusion

Aronia extract treatment increased skin cell proliferation and increased synthesis of type I collagen. On the other hand, this extract reduced MMP1 and MMP3 without affecting the stability of COL1A1 mRNA in the skin cell cultured in 2D environment. We investigated how this effect shows in a 3D environment using a dermal equivalent fabricated by a printing method. The change in mechanical properties due to collagen synthesis was observed in direct proportion to the concentration of Aronia extract, and the expression levels of *MMP1* and *MMP3* were inversely proportional to the concentration of Aronia extract. This study demonstrated that Aronia extract regulates collagen synthesis in dermal fibroblasts through the activation of COL1A1 transcription and a reduction in MMP-mediated type I collagen degradation.

## Data Availability

The data that support the findings of this study are available from the corresponding author upon reasonable request.
